# Psychological Distress and Health-Related Quality of Life in Public Sector Personnel

**DOI:** 10.3390/ijerph18041865

**Published:** 2021-02-14

**Authors:** Christina S. Malfa, Katerina Karaivazoglou, Konstantinos Assimakopoulos, Philippos Gourzis, Apostolos Vantarakis

**Affiliations:** 1 Department of Public Health, Medical School, University of Patras, 26504 Patras, Greece; kristamal@windowslive.com; 2 Psychiatric Clinic, Medical School, University of Patras, 26504 Patras, Greece; karaivaz@hotmail.com (K.K.); kassima@upatras.gr (K.A.); pgourzis@upatras.gr (P.G.)

**Keywords:** health-related quality of life, public health, work, stress, public personnel, SF-36, HADS, working conditions

## Abstract

Background: This study has assessed health-related quality of life (HRQoL) and psychological distress between public sector professional groups. Methods: Short Form-36 Health Survey (SF-36) and Hospital Anxiety and Depression Scale (HADS) were administered to healthcare personnel, schoolteachers, and municipality and regional personnel in the region of Western Greece. Mean scores on all SF-36 dimensions and HADS were compared among these professional groups as well as with the Greek national norms to assess if there were significant differences between our study sample and the general population. Results: Healthcare personnel reported significantly lower SF-36 scores than teachers and municipality employees. Women reported poorer HRQoL than men on all SF-36 dimensions. This overall low score for health care workers masks the fact that male health care workers, primarily medical doctors, actually scored better than women, primarily nurses and auxiliary personnel. Average mean scores on all SF-36 dimensions reported by nurses and auxiliary personnel in healthcare were considerably lower than the ones from employees in all other occupational types. The impact on HRQoL observed mainly in vitality, social functioning and mental health was important. Many participants have shown psychological burden and depression. Conclusions: The health inequalities among healthcare employees are significant. This study is important to suggest taking measures for improving the HRQoL of health workers.

## 1. Introduction

In modern societies, labor occupies a major part of people’s time and defines the quality of everyday living through type of occupation, income, working hours, routines, and professional relationships [1,2,3,4]. Professional status is associated with people’s well-being, while unemployment has been repeatedly linked to medical and psychological morbidity [5,6]. Similarly, work satisfaction is directly associated with overall life satisfaction [7]. There are several studies which reveal that professional insecurity and unstable working environment may lead to increased medical and psychological difficulties and adversely affect people’s well-being [6,8,9,10,11,12]. Certain professional groups, including healthcare and teaching personnel, appear even more vulnerable to work-related stress and professional burn-out and must cope with increased medical and psychosocial burden.

Stress is often aroused by stressors, stimuli in the environment that require an adaptive response from the individual. Stressors can be extra-organizational, organizational, task-related, or individual [13]. Responses to stressors have been called “eustress” (adaptive) or “distress” (nonadaptive) responses. Exposure to stressors may lead to negative health-related distress and eustress [14]. Occupational stress, and its symptoms such as feeling irritable, sleep disorders, hypertension, anxiety, and chronic cynicism, is a syndrome of emotional exhaustion, dissociation, and reduced personal self-confidence. Physical wellbeing refers to physical state of health, including calmness and energy; mental wellbeing refers to psychological health, including contentment, resilience, and peace of mind [14]. Significant research has been done suggesting that stress and its outcomes are influenced by individual factors, such as values, attitudes, perceptions, personalities, political skills, and motivations, as well as organizational factors, such as job design and organizational structure [15,16].

In recent years, there has been scientific interest in measuring HRQoL in several groups, including patients, caregivers, and even non-clinical cases such as specific social or professional groups [17,18]. The HRQoL was developed in medical sciences, focusing mainly on the assessment of physical and psychological health and social well-being [19,20], although, in its broadest sense, it characterized quality of life by focusing mainly on health assessment [20,21]. In general, it includes the general health, functional ability, physical symptoms, and emotional, cognitive, social, and general functioning of the individual [22,23]. There are a limited number of studies about HRQoL, involving specific occupational categories, such as health, teachers, and public sector personnel. Moreover, the investigation of the HRQoL of these occupational categories in Greece is negligible; therefore, this study is particularly interesting, since it will assess these professionals’ health level and cover part of this research gap.

The aim of our study was to evaluate the HRQoL and psychological distress of public sector professionals in Greece, and to investigate the factors affecting it. To our knowledge, this is the first study attempting to compare these parameters between these professional categories and delineate possible associations or differences. Mapping, accurately, the level of psychosocial functioning of people working in the public sector, in key domains of society, including health, education, and public administration, is of crucial importance and would give policy-makers useful insight in designing and implementing appropriate interventions to improve health quality of these professional groups.

## 2. Methods

The current study was conducted by the Department of Public Health in collaboration with the Psychiatric clinic of the Medical School of the University of Patras. The study protocol has been approved by the Institutional Review Board of Ethics of the University.

### 2.1. Sample

Our sample was public personnel, working in the region of Western Greece. The region includes three subregions, Achaia, Ileia, and Aitoloakarnania. Personnel were working in public health, public education, and employees within administrative sectors of municipal and regional governments. Only professional groups in the public sector were selected in the study, to exclude the impact of job insecurity. Sample was randomly selected. Only personnel that worked in the same job position for at least 2 years and were fluent in the Greek language were included in the study. In addition, cases of intellectual or physical disability were excluded to control for factors that could independently affect participants’ psychological functioning and HRQoL.

### 2.2. Instruments

All participants were asked to anonymously complete a detailed questionnaire comprising of 125 items. The first part of the questionnaire included questions about demographic characteristics, working conditions, health issues, habits, and life satisfaction. The second part included two instruments which are widely used to measure HRQoL and psychological distress. HRQoL was assessed with the Greek version of the Short Form-36 Health Survey (SF-36), which is a self-reported, generic HRQoL validated instrument. It includes 8 multi-item scales (36 items) that evaluate the extent to which an individual’s health limits his or her physical, emotional, and social well-being. More specifically, it covers 8 domains of HRQoL, namely physical functioning, role limitations due to physical problems, bodily pain, general health perception, vitality, social functioning, role limitations due to emotional problems, and mental health. Scores on each subscale range from 0 to 100, with higher scores indicating a better HRQoL result. The Greek version of the SF-36 provides population-based normative data which make possible the calculation of norm-based scores for each sub-scale. Norm-based scores below 50 indicate impaired functioning compared to the general population [18,24].

Psychological distress is a broadly used term that describes a state of emotional turmoil characterized by symptoms of anxiety and depression [25]. In the current study, the Greek version of the Hospital Anxiety and Depression Scale was used to measure anxious and depressive symptomatology reflecting participants’ level of psychological distress. This is a widely used psychometric tool, comprised of 14 items: 7 items for anxiety (HADS-A) and 7 items for depression (HADS-D). Each subscale is scored from 0–21. Higher scores indicate greater severity of depressive and anxious symptoms [26]. The last part of the questionnaire included 3 questions that evaluated the questionnaire as well as the whole administration process.

### 2.3. Ethics Approval and Consent to Participate

Our research was performed in accordance with the Declaration of Helsinki. The consent obtained from study participants was verbal. At the beginning of the questionnaire, an explanation paragraph was mentioned to explain to the participants about the research plan. The research protocol was approved from the Institutional Review Board of Ethics of the University of Patras.

### 2.4. Statistical Analysis

The study sample was divided into three groups, in which statistical analysis was conducted. Normality of distribution was analyzed with Kolmogorov–Smirnov. Descriptive and frequency statistics were used to compare demographic characteristics in tables, HADS, and SF-36 for each group. The statistical significance was tested with parametric (*t*-test and ANOVA) and non-parametric tests (Mann–Whitney and Kruskal–Wallis). Kruskal–Wallis was used to compare average scores from three groups. To analyze work conditions effect on scores in HADS and SF-36, Kruskal–Wallis was used. To analyze gender effect on scores in HADS and SF-36, Mann Whitney was used. *t*-tests for variables and Pearson Chi-Square tests for categorical variables were performed to assess the statistical significance of possible differences between groups. The differences among the groups were determined by applying ANOVA. All analyses were adjusted for possible confounding factors. For all analyses, a *p*-value of <0.05 (two-tailed) was considered statistically significant. The analyses were performed in SPSS 24 (IBM, Armonk, NY, USA).

## 3. Results

Five hundred and sixty (560) employees were approached to participate to the study, 436 of them (77.8%) responded to the questionnaires. Table 1 provides demographic characteristics, BMI, and smoking and alcohol use for each professional group and comparisons between groups. No statistical difference in the demographic parameters between the three groups of employees was recorded. Similarly, the three groups did not differ in BMI and smoking habits. The only significant difference reported was in alcohol use (*p* = 0.005). Administrative personnel reported greater weekly consumption of alcohol compared to healthcare (*p* = 0.009) and teaching personnel (*p* = 0.01).

The percentage of participants presenting clinically significant anxiety and depression levels, using a cut-off score of 8 for each HADS sub-scale, was assessed. One hundred and thirty-eight (59.7%) healthcare employees, 69 (61.1%) teachers, and 47 (51.1%) administrative employees reported clinically significant anxiety symptoms. Likewise, 199 (86.1%) healthcare employees, 93 (82.3%) teachers, and 79 (85.7%) administrative employees reported clinically significant depressive symptoms.

Subsequently, norm-based scores for each sub-scale of the SF-36 were calculated to determine the HRQoL domains that were most impaired in each professional category. For healthcare personnel, the greatest impairments were observed in the domains of vitality (45,97%), mental health (46.75%), general health (48.27%), and social functioning (45.43%). In the group of teachers, the greatest impairments were observed in vitality (49.42%) and mental health (49.92%), while in the group of administrative personnel, most participants scored below average only in the domain of mental health (49.74%). A Pearson Correlation Table has been prepared for the correlation of scores on the instruments used in the study and the demographic variables (Table 2).

Afterwards, ANOVA was performed to compare HADS-A, HADS-D, and SF-36 subscales mean scores between the three groups of employees (Table 3). This analysis revealed no significant difference in HADS-A and HADS-D scores. In contrast, significant differences between group differences emerged for all SF-36 subscales scores were recorded (Table 3). More specifically, healthcare employees reported significantly lower scores compared to teachers and administrative personnel in physical functioning (*p* < 0.001 and *p* = 0.004), role limitations due to physical problems (*p* < 0.001 and *p* < 0.001), bodily pain (*p* < 0.001 and *p* < 0.001), general health (*p* = 0.017 and *p* = 0.016), vitality (*p* < 0.001 and *p* < 0.001), social functioning (*p* = 0.004 and *p* = 0.001), and mental health (*p* = 0.002 and *p* = 0.012). In the domain of role limitations, due to emotional problems, healthcare workers appeared more impaired compared to administrative employees (*p* = 0.012), but not compared to teachers (*p* = 0.080). No significant differences emerged in any SF-36 subscale between teachers and administrative personnel.

## 4. Discussion

The current study constitutes one of the very few attempts to evaluate the differences in HRQoL and psychological distress of three working populations of public sector (healthcare, teaching, and administrative employees) in Western Greece. Public sector jobs were chosen to exclude the impact of uncertainty and instability of work on quality of life and distress.

In our study, no differences were found by age. Differences among the three groups were also insignificant for BMI, smoking habits, and demographic characteristics, which are parameters that could independently affect HRQoL and psychological functioning, thus confounding our results [27,28].

The main differences, among the different subgroups, concerned professional status, gender, and working conditions. Differences in the health status of the participants related to gender, showed similar patterns to those presented in other studies, and constituted an indication of the construct validity of the Greek version of SF-36. Men scored higher than women on all dimensions of SF-36. These findings are consistent with normative data from USA and Canada. However, gender-related differences in our study are larger than those observed elsewhere [29] but are like other studies in Greece [30]. These gender differences, to some extent, seem to reflect the disadvantageous position of woman in a predominantly male societal structure of professional life. Perhaps this is even more the case for Greece, which is a Mediterranean country where women’s social position is traditionally worse than that of men. Nevertheless, it is possible the great magnitude of these gender differences observed in our study reflect a confounding factor as a result of professional differences between men (who were mostly medical doctors) and women (who were mostly nurses and auxiliary personnel) [31].

Our data reveals some important issues. The first issue is that health inequalities among the employees in different professions in public sector, concerned healthcare employees. The comparisons suggested that healthcare personnel were more affected in all sub-scales of HRQoL compared to teaching and administrative personnel, and this is consistent with previous literature [32,33,34,35]. Our finding that healthcare personnel’s QoL was affected in the domains of general health, vitality, mental health, and social functioning corroborates previous research in Greece [31] and abroad [36]. There were no significant differences between groups in anxiety and depression scores. The differences observed on the various subscales of SF-36 and HADS between the different occupations were consistent with health research arguing about the impact of social gradient within the workplace and occupational hierarchy on health and well-being of people [36]. Unfortunately, the existing literature on HRQoL of healthcare professionals is not so abundant. Most studies assessing the health status of healthcare professionals have used other generic health instruments (mostly General Health Questionnaire–GHQ), and therefore a direct comparison between their findings and the results of the present study could not be made.

A major issue was the health inequalities among health care employees in Greek hospitals, which was the main target group in our study. Our results show that the health employees do not constitute a homogenous group of employees with similar health status and HRQoL. In a similar study with nurses in New Zealand [37], higher mean scores were reported than our study. Nurses’ poor health status and HRQoL primarily showed the difficulties they face in their work. They work a highly stressful and demanding profession which is not very well rewarded, as reported in several studies [38,39,40,41]. The auxiliary personnel (although they were low in number in our study) seem to have the worst health status and poorer HRQoL compared to other studies [42,43,44]. We reported the lowest scores on all SF-36 dimensions, apart from that of mental health where nurses scored worse.

Our studied personnel could be divided into clearly distinct professional groups, namely, one group with a good state of health consisting mainly of medical doctors, teachers, and administrative and technical staff (mostly engineers); a second professional group consisting of administrative personnel whose scores were satisfactory; and a third one consisting of nurses and auxiliary staff with the lowest score. The first group showed the best characteristics as the administrative personnel (public sector) are between medical doctors and technical personnel and the nurses and auxiliary staff) on almost all the SF-36 dimensions.

Most participants reported symptoms of depression and anxiety, independently of professional category. This finding calls for special attention. There are several studies reporting that healthcare workers, especially physicians, nurses, and emergency department personnel, exhibit increased rates of depressive and anxious symptomatology. In a recent study, in Greek oncology nurses [45], 11% reported clinically significant anxiety symptoms, while 24% and 10% of emergency unit nurses reported severe depressed or anxious mood, respectively. The lower rates of psychological distress observed in those studies compared to ours might be attributed to the use of a different assessment instrument with different sensitivity compared to HADS. Economou et al. (2013) [46] reported that there was a 2.5 increase in major depression prevalence in Greece between 2008 and 2011, which was the period before and after the financial crisis of 2009. In addition, these researchers suggested that the increase of depression prevalence is continuous, and major depression rates are expected to be even higher in the present era. In this respect, our results reflect the greatest psychological burden imposed onto Greek society in the years of crisis. In a recent Egyptian study, 37.5% of working women reported clinically significant depressive symptoms [47]. In a wide-scale study in China, researchers found that healthcare personnel, teachers, and civil servants exhibit greater mental health impairment compared to manual workers [48]. Similarly, another study showed that teachers and healthcare personnel are at increased risk of common mental disorders, mostly depression and anxiety [49]. Wieclaw (2006) [50] reported that teaching and healthcare services are associated with increased risk of anxiety and depression disorders.

Finally, the differences between our study results and the national norms of Greece are important. The study groups in our study scored considerably lower on the 8 domains of the SF-36 than their counter partners in the general population in Greece, as well as other studies from Western Europe and North America. Our scores are lower than the Canadian, UK, and Swedish norms [50].

The present study has few limitations. Firstly, the study population is not representative for the country. Another important point is that the comparison between the scores of this study and other national norms are problematic since it is not age-adjusted, and the study sample is not necessarily representative of the Greek general population. Consequently, any conclusion drawn from this comparison should be treated with caution. Another limitation is the small number of mainly young participants, which put into question the value of the observed health differences between the age groups of the sample, although this is due to the characteristic of these professional groups in Greece. Finally, the difference in samples of the three categories is important and may affect the conclusions between the groups. Notwithstanding the acknowledged limitations, the results of the study, when examined overall, are sufficiently disturbing enough to suggest that further work in this area is needed to inform the design of policy to improve what appears to be a serious problem in Greece’s health care system.

To conclude, there are major health inequalities among the employees, especially for healthcare employees. This study is important for public health policy and for taking measures to improving the Quality of life (QOL) of health workers. There is a necessity for taking public health policy measures to improve the HRQoL of health workers. These health inequalities underscore the need for interventions to tackle them and initiatives to support healthcare employees and especially nurses, women, and young and low professional status workers in the Greek hospitals.

## Figures and Tables

**Table 1:** Demographic and other characteristics of the participants by professional group.

Characteristic	Healthcare Personnel (1)	Teaching Personnel (2)	Administrative Personnel (3)	*p*-Value
N	231	113	92	
Gender, male (%)	53 (23.0)	31 (27.4)	31 (33.7)	0.141
Age, mean (SD)	42.49 (8.64)	42.80 (9.88)	43.36 (7.66)	0.736
Marital status				
Single (%) Married (%) Widowed (%) Divorced (%) Co-living (%) N/A (%)	54 (23.5) 148 (64.3) 3 (1.3) 15 (6.5) 9 (3.9) 1 (0.4)	26 (23.0) 76 (67.3) 2 (1.8) 3 (2.7) 4 (3.5) 2 (1.8)	26 (28.3) 56 (60.9) 2 (2.2) 8 (8.7) 0 (0.0) 0 (0.0)	0.357
Parenthood, yes (%)	149 (65.1)	77 (68.1)	58 (63)	0.736
Years of education, mean (SD)	15.98 (1.95)	16.24 (1.31)	16.20 (2.31)	0.422
Years of work, mean (SD)	14.79 (9.38)	15.11 (8.77)	13.55 (8.03)	0.414
Chronic disease, yes (%)	83 (36.4)	28 (25.5)	22 (23.9)	0.121
BMI, mean (SD)	24.64 (4.07)	24.64 (3.39)	25.09 (3.77)	0.676
Smoking, yes (%)	69 (30.3)	31 (27.4)	29 (31.9)	0.721
Alcohol units/wk, mean (SD)	1.61 (3.00)	1.46 (1.92)	2.72 (3.77)	0.005 *

* Correlation is significant at the 0.05 level (2-tailed).

**Table 2:** Pearson Correlation of Anxiety, depression and health-related quality of life (HRQoL) scores (means and SD) by demographic variables.

	Gender	BMI	City
HADS-A	−0.169 **	−0.039	−0.010
HADS-D	−0.087	−0.156 **	0.022
Physical Functioning	0.101 *	−0.164 **	−0.020
Role Physical	0.103 *	−0.102	−0.032
Bodily pain	0.193 **	−0.001	0.008
General Health	0.169 **	−0.033	−0.052
Vitality	0.222 **	0.044	0.091
Social Functioning	0.218 **	0.001	0.024
Role emotional	0.154 **	0.037	−0.010
Mental Health	0.155 **	0.015	0.035

** Correlation is significant at the 0.01 level (2-tailed) * Correlation is significant at the 0.05 level (2-tailed).

**Table 3:** Anxiety, depression and HRQoL scores (means and SD) by professional category and between-group comparisons.

Scale	Healthcare Personnel (1)	Teaching Personnel (2)	Administrative Personnel (3)	*p* Values		
				1 vs. 2	1 vs. 3	2 vs. 3
HADS-A	8.48 (2.49)	8.45 (2.37)	7.95 (2.48)	>0.99	0.228	0.445
HADS-D	9.38 (2.06)	9.19 (2.01)	9.21 (1.98)	>0.99	>0.99	>0.99
Physical Functioning	81.74 (20.52)	90.77 (12.05)	89.28 (17.52)	<0.001 *	0.004 *	0.871
Role Physical	73.24 (35.15)	87.05 (25.99)	87.36 (24.83)	<0.001 *	<0.001 *	>0.99
Bodily pain	73.80 (24.70)	85.23 (17.61)	85.91 (16.30)	<0.001 *	<0.001 *	0.988
General Health	63.56 (16.01)	68.47 (13.76)	68.88 (15.54)	0.017 *	0.016 *	>0.99
Vitality	57.60 (18.59)	65.22 (14.95)	67.64 (15.14)	<0.001 *	<0.001 *	0.590
Social Functioning	69.21 (25.31)	77.82 (21.63)	79.26 (20.94)	0.004 *	0.001 *	0.950
Role emotional	72.56 (35.87)	80.95 (31.23)	83.88 (29.55)	0.080	0.012 *	0.871
Mental Health	61.54 (17.25)	68.14 (15.16)	67.47 (15.74)	0.002 *	0.012 *	>0.99

HADS-A: Hospital Anxiety Depression Scale-Anxiety Subscale; HADS-D: Hospital Anxiety Depression Scale-Depression Subscale; * Significant at the *p* < 0.05 level, all other comparisons were not significantly different.

## Data Availability

The datasets used and/or analyzed during the current study are available from the corresponding author on reasonable request.

## Abbreviations

HRQoL	Health-related quality of life
GHQ	General Health Questionnaire
BMI	Body Mass index
QOL	Quality of Life
